# Accuracy of the Final Growth Ultrasound in Predicting Birthweight Centile in Women With Class III Obesity: A Retrospective Cohort Study

**DOI:** 10.7759/cureus.119371

**Published:** 2026-11-09

**Authors:** Supreet Mahurkar, Shermin Riaz, Bojana Salovic, Soneya Chowdhury, Anjali Anjali, Sajitha Parveen

**Affiliations:** 1 Obstetrics and Gynaecology, Aneurin Bevan University Health Board, Gwent, GBR; 2 Emergency Medicine, Midland Metropolitan University Hospital, Smethwick, GBR; 3 Obstetrics and Gynaecology, The Grange University Hospital, Cwmbran, GBR

**Keywords:** birthweight centile, body mass index, class iii obesity, estimated fetal weight, fetal growth restriction, large for gestational age, pregnancy outcomes, ultrasound surveillance

## Abstract

Introduction

This study evaluated agreement between final third-trimester ultrasound estimated fetal weight (EFW) centile and subsequent birthweight centile in singleton pregnancies among women with class III obesity (body mass index (BMI) ≥40 kg/m²). Secondary objectives were to assess antenatal detection of small-for-gestational-age (SGA) and large-for-gestational-age (LGA) infants and associations of maternal BMI and scan-to-delivery interval with centile prediction error.

Methods

We conducted a retrospective cohort study of singleton pregnancies in women with booking BMI ≥40 kg/m². Agreement between final ultrasound EFW centile and birthweight centile was assessed using centile differences, correlation, SGA/appropriate-for-gestational-age (AGA)/LGA categorical agreement, and weighted Cohen’s kappa. Secondary analyses assessed diagnostic performance for SGA and LGA and associations of maternal BMI and scan-to-delivery interval with centile error.

Results

A total of 245 pregnancies had complete data. Mean maternal BMI was 44.8 kg/m², and mean scan-to-delivery interval was 14.4 days. EFW centile was positively correlated with birthweight centile (r = 0.732, p < 0.001). The median absolute centile difference was 13.1 centiles, and the mean signed difference was +3.6 centiles (95% confidence interval (CI) 1.0-6.2). Overall SGA/AGA/LGA category agreement was 82.4% (weighted κ = 0.502). Sensitivity was 26.9% for SGA and 46.9% for LGA, with specificities of 99.5% and 97.2%, respectively. Absolute centile error did not differ significantly across BMI or scan-to-delivery interval groups.

Conclusions

Final third-trimester ultrasound EFW centile was positively correlated with birthweight centile in women with class III obesity, but clinically important individual discrepancies remained. The final scan had high specificity but low sensitivity for identifying infants subsequently born SGA or LGA. Increasing BMI within the class III obesity range and scan-to-delivery interval were not significantly associated with greater centile error.

## Introduction

Maternal obesity is a major public health challenge and one of the most common medical complications encountered in contemporary obstetric practice. Women with class III obesity (body mass index (BMI) ≥40 kg/m²) are at increased risk of gestational diabetes, hypertensive disorders of pregnancy, operative delivery, postpartum haemorrhage, stillbirth, and adverse neonatal outcomes [1-4].

Maternal obesity is also associated with abnormalities of fetal growth. Both fetal growth restriction and fetal macrosomia may occur in pregnancies complicated by severe obesity. Accurate antenatal identification of abnormal fetal growth is therefore important to facilitate appropriate surveillance, counselling, and delivery planning [4,5].

Ultrasound-estimated fetal weight (EFW) remains central to third-trimester fetal growth assessment. In the United Kingdom, fetal growth surveillance is frequently expressed using centiles, and many clinical decisions are influenced by a fetus’s position on customised growth charts [6]. Increased maternal adiposity may reduce ultrasound image quality and can make assessment of fetal size more challenging [1]. Previous studies have evaluated the utility of ultrasound for identifying fetal growth disorders in obese pregnancies [7]. Ultrasound assessment also plays a key role in the antenatal identification and management of fetal growth restriction, which remains an important contributor to perinatal morbidity and mortality. Professional guidance recommends the use of fetal biometry and EFW to support surveillance and clinical decision-making when fetal growth restriction is suspected [8].

Several studies have demonstrated that sonographic estimation of fetal weight remains reasonably accurate in women with obesity [9-13]. However, much of the existing literature has focused on absolute weight error or percentage weight error rather than agreement between antenatal growth centiles and actual birthweight centiles [9-13]. As clinical decision-making is commonly guided by fetal growth centiles rather than estimated weight alone, assessment of centile agreement may provide information that is directly relevant to routine practice.

Uncertainty also remains regarding the ability of ultrasound surveillance to identify fetuses at the extremes of growth. Failure to recognise small-for-gestational-age (SGA) or large-for-gestational-age (LGA) fetuses may result in missed opportunities for additional surveillance or delivery planning. Studies evaluating ultrasound performance in obese populations have reported variable detection rates for SGA and LGA infants [7,12,13].

The primary aim of this study was to evaluate agreement between the final antenatal EFW centile and birthweight centile in women with class III obesity. Secondary objectives were to assess antenatal detection of SGA and LGA infants, examine whether increasing BMI within the class III obesity range was associated with centile error, and investigate whether scan-to-delivery interval influenced predictive accuracy.

## Materials and methods

Study design and setting

This retrospective cohort study was conducted within the maternity services of Aneurin Bevan University Health Board, Wales, United Kingdom. Data were retrospectively collected from electronic maternity records for eligible women who delivered between 1 January 2025 and 31 January 2026. The project used routinely collected clinical data and anonymised patient information.

Study population

Women were eligible if they had a singleton pregnancy, booking BMI ≥40 kg/m², a documented third-trimester growth ultrasound examination, available final ultrasound EFW centile and birthweight centile data, and a live birth. Pregnancies were excluded for multiple gestation, major fetal anomaly, intrauterine fetal death, or incomplete data required for the primary analysis.

Data collection

Maternal variables included BMI, gravidity, and parity. Pregnancy variables included gestational age at the final growth ultrasound, EFW, EFW centile, gestational age at delivery, scan-to-delivery interval, birthweight, birthweight centile, and mode of delivery. EFW centiles and birthweight centiles were extracted directly from BadgerNet, the electronic maternity record system used at the study centre. The recorded centiles were generated using Gestation Related Optimal Weight (GROW) 2.0 (Customisable), version 2.0.6.3 (Gestation Network Ltd., Birmingham, United Kingdom), a customised growth-chart system [6]. Centiles were obtained from routinely documented maternity records and were not independently recalculated for this study.

Outcomes

The primary outcome was agreement between final ultrasound EFW centile and birthweight centile. Absolute centile difference was defined as $|\text{EFW centile} - \text{birthweight centile}|$. Signed centile difference was defined as $\text{EFW centile} - \text{birthweight centile}$, such that positive values represented an antenatal centile higher than the subsequent birthweight centile. For descriptive comparison with the original study plan, absolute differences were grouped as <10, 10-<20, 20-<30, 30-<40, 40-<50, and ≥50 centiles. These thresholds were prespecified descriptive categories and were not intended as validated clinical limits of acceptable agreement.

SGA was defined as <10th centile, appropriate-for-gestational-age (AGA) as 10th-90th centile, and LGA as >90th centile. Diagnostic performance of the final EFW category for SGA and LGA at birth was assessed using sensitivity, specificity, positive predictive value (PPV), and negative predictive value (NPV), with 95% confidence intervals (CIs). Overall categorical agreement was also assessed.

Exploratory analyses examined absolute centile error across BMI groups (40-44.9, 45-49.9, and ≥50 kg/m²) and scan-to-delivery intervals (0-7, 8-14, and >14 days). Because direct comparison of EFW in grams with birthweight becomes increasingly confounded by true fetal growth as the scan-to-delivery interval lengthens, conventional percentage weight error was examined only as a sensitivity analysis among scans performed within seven days of delivery.

Statistical analysis

Continuous variables are presented as means with standard deviations or medians with interquartile ranges where appropriate; categorical variables are presented as frequencies and percentages. Pearson’s correlation coefficient assessed the linear association between EFW and birthweight centiles. Agreement was characterised using signed and absolute centile differences, a centile-difference plot, and SGA/AGA/LGA categorical agreement. Weighted Cohen’s kappa with quadratic weighting was calculated for the ordered three-category classification. The 95% CI for overall categorical agreement was calculated using the Wilson method. The 95% CI for weighted Cohen’s kappa was estimated using 10,000 paired bootstrap resamples with the percentile method and a fixed random seed of 2026. Wilson 95% CIs were calculated for diagnostic performance measures. Kruskal-Wallis tests compared absolute centile error across BMI and scan-to-delivery interval groups, and Spearman correlation assessed continuous associations. Exploratory multivariable ordinary least-squares linear regression included BMI and scan-to-delivery interval as predictors of absolute centile error; regression coefficients, 95% CIs and individual p-values were calculated. Statistical significance was defined as a two-tailed p-value <0.05. Analyses were performed using Python 3.13 (Python Software Foundation, Wilmington, Delaware, USA) with pandas, SciPy, statsmodels, and scikit-learn. A complete-case analysis was performed. Records missing variables required for the primary analysis were excluded, and no missing values were imputed.

Ethical considerations

This study utilised anonymised routinely collected clinical data. No patient-identifiable information was analysed. The project was undertaken as a retrospective service evaluation and complied with local information-governance requirements.

## Results

Patient characteristics

A total of 258 pregnancy records were screened, of which 13 (5.0%) were excluded because of incomplete data required for the primary analysis. The final cohort comprised 245 pregnancies (95.0%) with complete paired ultrasound EFW and birthweight centile data. Mean maternal BMI was 44.8 kg/m² (median 43.5 kg/m²). Median gravidity was 2 and median parity was 1. Mean gestational age at the final growth scan was approximately 37+4 weeks and mean gestational age at delivery was approximately 39+5 weeks. The mean scan-to-delivery interval was 14.4 days (median 14 days). Mean EFW centile was 49.2, compared with a mean birthweight centile of 45.5.

Baseline maternal and pregnancy characteristics are summarised in Table 1.

**Table 1:** Baseline Maternal and Pregnancy Characteristics BMI, body mass index; EFW, estimated fetal weight; GA, gestational age

Characteristic	Value
Number of pregnancies	245
Mean BMI (kg/m²)	44.8
Median BMI (kg/m²)	43.5
Median Gravidity	2
Median Parity	1
Mean GA at Final Growth Scan	37+4 weeks
Mean GA at Delivery	39+5 weeks
Mean Scan-to-Delivery Interval	14.4 days
Median Scan-to-Delivery Interval	14 days
Mean EFW Centile	49.2
Mean Birthweight Centile	45.5
Mean Absolute Centile Difference	16.0
Median Absolute Centile Difference	13.1

Agreement between ultrasound EFW centile and birthweight centile

A positive correlation was observed between EFW centile and birthweight centile (r = 0.732, p < 0.001; R² = 0.5355) (Figure 1).

**Figure 1:**
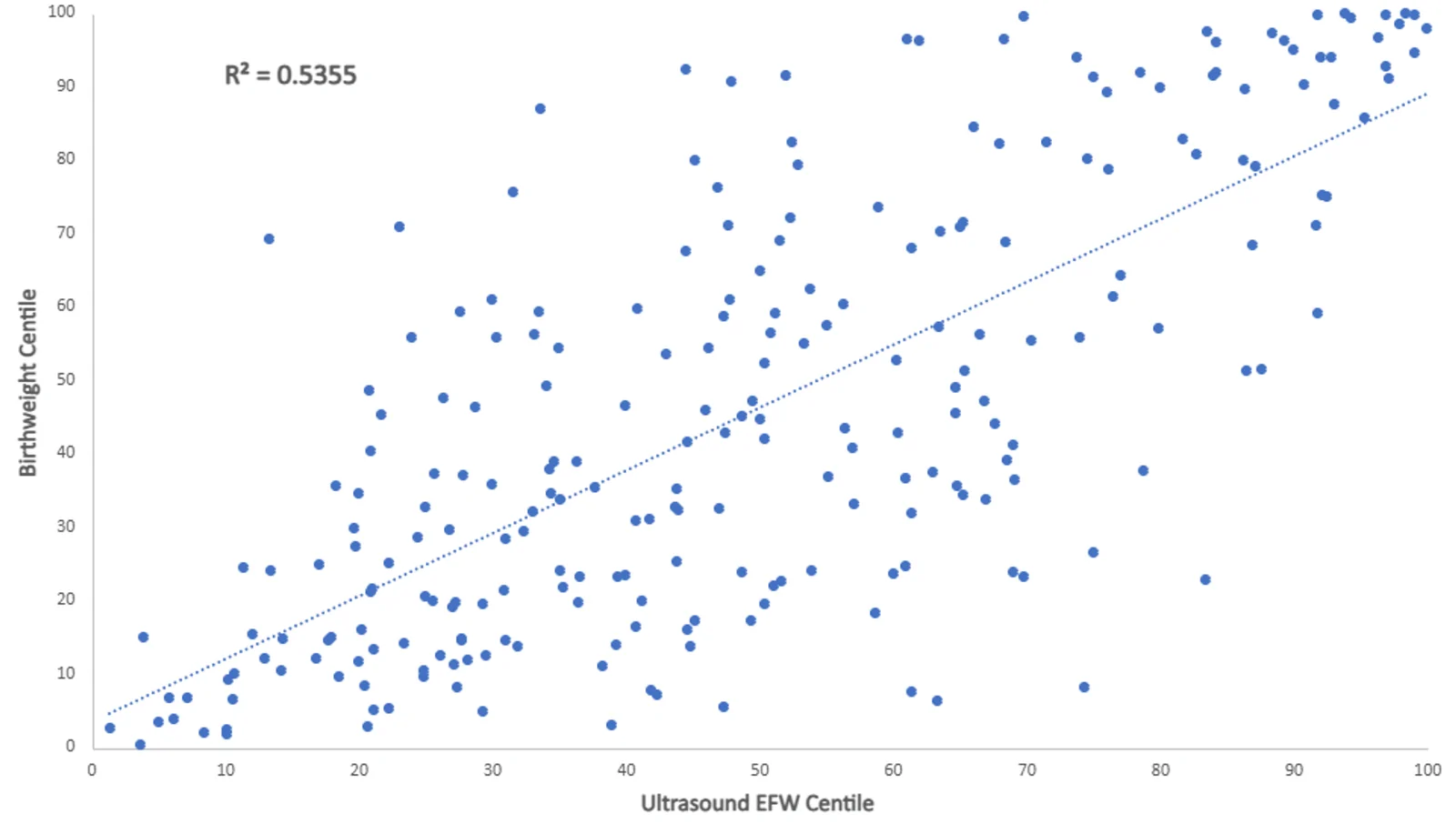
Correlation Between Ultrasound EFW Centile and Birthweight Centile Scatter plot demonstrating the relationship between ultrasound estimated fetal weight (EFW) centile and birthweight centile in women with class III obesity. A strong positive correlation was observed between antenatal EFW centile and birthweight centile (r = 0.732; R² = 0.5355).

The mean absolute centile difference between ultrasound EFW centile and birthweight centile was 16.0 centiles, while the median absolute centile difference was 13.1 centiles.

Agreement categories are presented in Table 2. Overall, 103 pregnancies (42.0%) demonstrated agreement within 10 centiles of the eventual birthweight centile. Agreement within 20 centiles was observed in 173 pregnancies (70.6%), while 206 pregnancies (84.1%) demonstrated agreement within 30 centiles.

**Table 2:** Agreement Between Final Ultrasound EFW Centile and Birthweight Centile EFW, estimated fetal weight

Absolute Centile Difference	n (%)
<10	103 (42.0)
10-<20	70 (28.6)
20-<30	33 (13.5)
30-<40	23 (9.4)
40-<50	10 (4.1)
≥50	6 (2.4)
Total	245 (100)

Twenty-three pregnancies (9.4%) demonstrated an absolute centile difference of 30-<40 centiles, 10 pregnancies (4.1%) demonstrated a difference of 40-<50 centiles, and six pregnancies (2.4%) demonstrated a discrepancy greater than or equal to 50 centiles.

Categorical agreement and detection of growth abnormalities

Overall, 202 of 245 pregnancies (82.4%, 95% CI 77.2%-86.7%) were classified into the same SGA/AGA/LGA category antenatally and at birth. Quadratic-weighted Cohen’s kappa was 0.502 (95% CI 0.363-0.624), indicating moderate agreement beyond chance. Among 216 fetuses classified as AGA on the final ultrasound, 36 (16.7%) were subsequently at a birthweight extreme: 19 were SGA and 17 were LGA (Table 3).

**Table 3:** Cross-Classification of Final Ultrasound EFW Category and Birthweight Category SGA, small-for-gestational-age; AGA, appropriate-for-gestational-age; LGA, large-for-gestational-age; EFW, estimated fetal weight

Final ultrasound category	Birth SGA	Birth AGA	Birth LGA
SGA	7	1	0
AGA	19	180	17
LGA	0	6	15

Twenty-six infants (10.6%) were born SGA; seven were identified as SGA on the final scan and 19 were not, giving a sensitivity of 26.9% and specificity of 99.5%. Thirty-two infants (13.1%) were born LGA; 15 were identified as LGA antenatally and 17 were not, giving a sensitivity of 46.9% and specificity of 97.2% (Table 4).

**Table 4:** Diagnostic Performance of the Final Ultrasound Category for SGA and LGA at Birth SGA, small-for-gestational-age; LGA, large-for-gestational-age; PPV, positive predictive value; NPV, negative predictive value

Outcome	Sensitivity (95% CI)	Specificity (95% CI)	PPV (95% CI)	NPV (95% CI)
SGA	26.9% (13.7-46.1)	99.5% (97.5-99.9)	87.5% (52.9-97.8)	92.0% (87.8-94.8)
LGA	46.9% (30.9-63.6)	97.2% (94.0-98.7)	71.4% (50.0-86.2)	92.4% (88.2-95.2)

Association with maternal BMI

Mean absolute centile difference increased descriptively from 15.4 centiles at BMI 40-44.9 kg/m² to 17.7 centiles at BMI ≥50 kg/m², but this difference was not statistically significant (Kruskal-Wallis p = 0.511) (Table 5). BMI was not significantly correlated with absolute centile error (Spearman ρ = 0.089, p = 0.165).

**Table 5:** Absolute Centile Difference According to Maternal BMI Subgroup

BMI group (kg/m²)	n	Mean absolute centile difference	Median absolute centile difference
40-44.9	157	15.4	12.9
45-49.9	59	16.9	14.6
≥50	29	17.7	11.6

Impact of scan-to-delivery interval

Absolute centile error was similar across scan-to-delivery interval groups (Kruskal-Wallis p = 0.937), with no significant continuous association between scan-to-delivery interval and absolute centile error (Spearman ρ = -0.039, p = 0.545) (Table 6). In an exploratory multivariable linear regression model, neither maternal BMI (β = 0.185, 95% CI -0.201 to 0.571, p = 0.346) nor scan-to-delivery interval (β = -0.045, 95% CI -0.262 to 0.172, p = 0.682) was significantly associated with absolute centile error (model R² = 0.005).

**Table 6:** Absolute Centile Difference According to Scan-to-Delivery Interval

Scan-to-delivery interval	n	Mean absolute centile difference	Median absolute centile difference
0-7 days	57	16.4	14.0
8-14 days	66	15.6	13.7
>14 days	122	16.1	11.8

Sensitivity analysis: conventional weight error within seven days of delivery

Among the 57 pregnancies with ultrasound performed within seven days of delivery, mean percentage error (EFW - birthweight, divided by birthweight) was -2.2%, and mean absolute percentage error was 6.7%. EFW was within ±10% of birthweight in 73.7% and within ±15% in 94.7% of these pregnancies. This restricted analysis reduces, but does not eliminate, the effect of true fetal growth between ultrasound and birth.

## Discussion

In this retrospective cohort of 245 women with class III obesity, final ultrasound EFW centile was positively correlated with subsequent birthweight centile (r = 0.732). However, correlation should not be interpreted as agreement. At the individual level, the median absolute difference was 13.1 centiles and the mean absolute difference was 16.0 centiles. The mean signed difference of +3.6 centiles indicated a small systematic tendency for the final ultrasound centile to exceed the subsequent birthweight centile.

The categorical analysis provides a clinically intuitive assessment of performance. Overall SGA/AGA/LGA classification agreement was 82.4%, with a weighted kappa of 0.502. Importantly, 36 of 216 fetuses classified as AGA on the final ultrasound were subsequently born either SGA or LGA. The final scan therefore performed substantially better at ruling in than ruling out the extreme growth categories: specificity was 99.5% for SGA and 97.2% for LGA, whereas sensitivity was only 26.9% and 46.9%, respectively.

The increasing prevalence of maternal obesity has highlighted the importance of reliable antenatal surveillance. Concerns have long existed that maternal adiposity may impair image quality and reduce the accuracy of fetal biometry. Previous studies, however, have generally found that sonographic EFW remains reasonably accurate across increasing BMI categories [9-13]. Ahmadzia et al. reported similar mean absolute percentage error across BMI categories in morbidly obese women [9], while Gevaerd Martins et al. found no significant deterioration in sonographic weight estimation with increasing BMI when measurements were performed by trained sonographers [10]. A large multicentre study by Dittkrist et al. similarly found no significant reduction in the proportion of EFW estimates within ±10% of birthweight among women with obesity [11].

A notable strength of the present study is its centile-based approach. Conventional studies of EFW accuracy generally compare estimated and actual weight in grams. When the final growth scan precedes delivery by days or weeks, however, part of the difference in grams reflects genuine fetal growth rather than measurement error. Centile comparison partially addresses this problem by relating fetal size to gestational age at each assessment and is also clinically relevant because SGA and LGA classifications are centile-based. Nevertheless, centiles are transformed, bounded measures and equal numerical differences do not necessarily represent equal differences in fetal weight across the distribution; centile agreement should therefore complement rather than replace conventional weight-error analyses.

The poor sensitivity for SGA is particularly important clinically. Dude et al. reported similarly poor sensitivity for SGA detection in obese nulliparous women (26.3%) despite very high specificity [7], closely resembling the pattern observed in the present cohort. This supports caution when a single apparently normal final EFW centile is used to exclude SGA.

Notably, this finding contrasts with a 2025 analysis of the National Institute of Child Health and Human Development (NICHD) Fetal Growth Studies cohort, which reported higher diagnostic accuracy for SGA classification in overweight/obese women than in women of normal weight (area under the receiver operating characteristic curve (AUC) 0.91 vs. 0.84, p = 0.004) [12]. Differences in study population, BMI range, growth standards, gestational-age windows, and scan-to-delivery interval may contribute to this discrepancy. The comparison emphasises that diagnostic performance may vary by clinical context and methodology rather than following a single pattern across all populations with obesity.

In the present cohort, increasing BMI within the class III obesity range was not significantly associated with greater absolute centile error. Although mean absolute error rose descriptively across BMI strata, from 15.4 centiles at BMI 40-44.9 kg/m² to 17.7 centiles at BMI ≥50 kg/m², the difference was not statistically significant. This finding is consistent with recent evidence suggesting that maternal BMI itself is not a major independent determinant of sonographic EFW precision. Granfors et al., in a population-based study of 31,521 pregnancies with ultrasound within two days of delivery, found that maternal BMI was not associated with reduced EFW precision, whereas fetal characteristics - particularly severe SGA - were more influential [14].

Further recent evidence supports the relative robustness of sonographic estimation across BMI strata. Krencel et al. compared clinical and sonographic fetal-weight estimation and found that sonographic accuracy remained stable as BMI increased, whereas clinical estimation became less accurate; maternal BMI was not independently associated with >20% error in sonographic EFW [15]. These findings support the use of ultrasound rather than clinical palpation when fetal-weight estimation is required in women with elevated BMI, while also emphasising that ultrasound remains an estimate rather than a direct measurement.

Scan-to-delivery interval was not significantly associated with absolute centile error in this cohort. Mean absolute centile differences were 16.4, 15.6, and 16.1 centiles for intervals of 0-7, 8-14, and >14 days, respectively. This differs from direct gram-based comparison, for which longer intervals inevitably incorporate true fetal growth. In the restricted subgroup scanned within seven days of delivery, mean absolute percentage error was 6.7% and 73.7% of estimates were within ±10% of birthweight, broadly consistent with published EFW accuracy data in obese populations [9,11].

The study has several limitations. Its retrospective, single-health-board design introduces potential selection bias and limits generalisability. There was no normal-BMI comparison group; therefore, the study cannot determine whether class III obesity itself causes the observed discrepancies. The numbers of SGA and LGA infants were relatively small, resulting in wide confidence intervals around sensitivity and PPV estimates. Potential confounders, including diabetes status, fetal sex, presentation, individual sonographer, and the biometric formula used for each scan, were not available for analysis. In addition, GROW centiles are customised, and the results may differ with alternative fetal and neonatal growth standards. The centile-difference limits of agreement should be interpreted cautiously because centiles are bounded and nonlinear.

Strengths include a real-world cohort specifically comprising women with class III obesity, complete paired centile data for 245 pregnancies, analysis of both continuous centile discrepancy and clinically meaningful SGA/AGA/LGA classification, and formal reporting of diagnostic performance with confidence intervals. The additional analysis across BMI and scan-to-delivery intervals also helps distinguish the effects of severe maternal obesity and timing from the intrinsic limitations of fetal-size prediction.

## Conclusions

Final third-trimester ultrasound EFW centile demonstrated moderate categorical agreement with subsequent birthweight classification in women with class III obesity. EFW centile was positively correlated with birthweight centile; however, clinically important individual discrepancies remained, particularly at the extremes of fetal growth. The final ultrasound demonstrated high specificity but relatively low sensitivity for identifying infants subsequently born SGA or LGA. Increasing BMI within the class III obesity range and scan-to-delivery interval were not significantly associated with greater centile error in this cohort. These findings support continued use of ultrasound for fetal growth assessment while emphasising that a single apparently normal EFW centile does not reliably exclude an infant who will ultimately be SGA or LGA. Further studies incorporating comparator BMI groups, fetal characteristics, and standardised short scan-to-delivery intervals would help clarify whether severe maternal obesity independently influences centile prediction.
